# Neighbourhood socioeconomic disadvantage and fruit and vegetable consumption: a seven countries comparison

**DOI:** 10.1186/s12966-015-0229-x

**Published:** 2015-05-22

**Authors:** Kylie Ball, Karen E. Lamb, Claudia Costa, Nicoleta Cutumisu, Anne Ellaway, Carlijn B. M. Kamphuis, Graciela Mentz, Jamie Pearce, Paula Santana, Rita Santos, Amy J. Schulz, John C. Spence, Lukar E. Thornton, Frank J. van Lenthe, Shannon N. Zenk

**Affiliations:** Centre for Physical Activity and Nutrition Research, School of Exercise and Nutrition Sciences, Deakin University, 221 Burwood Highway, Melbourne, VIC 3125 Australia; Centre of Studies on Geography and Spatial Planning (CEGOT), University of Coimbra, Coimbra, Portugal; The Research Center of the University of Montreal Hospital Centre (Centre de Recherche du Centre hospitalier de l’Université de Montréal - CRCHUM) & The Department of Social and Preventive Medicine, School of Public Health, University of Montreal, Montreal, Canada; Medical Research Council Social and Public Health Sciences Unit, University of Glasgow, Glasgow, UK; Erasmus University Medical Centre, Rotterdam, Netherlands; University of Michigan, Ann Arbor, MI USA; Centre for Research on Environment, Society & Health, School of GeoSciences, University of Edinburgh, Edinburgh, UK; Department of Geography, Faculty of Humanities, University of Coimbra, Coimbra, Portugal; Centre for Health Economics, University of York, York, United Kingdom; University of Alberta, Edmonton, Canada; University of Illinois, Chicago, IL USA

**Keywords:** Diet, Fruit, Vegetables, Socioeconomic Status, Neighbourhood, International

## Abstract

**Background:**

Low fruit and vegetable consumption is a risk factor for poor health. Studies have shown consumption varies across neighbourhoods, with lower intakes in disadvantaged neighbourhoods. However, findings are inconsistent, suggesting that socio-spatial inequities in diet could be context-specific, highlighting a need for international comparisons across contexts.

This study examined variations in fruit and vegetable consumption among adults from neighbourhoods of varying socioeconomic status (SES) across seven countries (Australia, Canada, Netherlands, New Zealand, Portugal, Scotland, US).

**Methods:**

Data from seven existing studies, identified through literature searches and knowledge of co-authors, which collected measures of both neighbourhood-level SES and fruit and vegetable consumption were used. Logistic regression was used to examine associations between neighbourhood-level SES and binary fruit and vegetable consumption separately, adjusting for neighbourhood clustering and age, gender and education. As much as possible, variables were treated in a consistent manner in the analysis for each study to allow the identification of patterns of association within study and to examine differences in the associations across studies.

**Results:**

Adjusted analyses showed evidence of an association between neighbourhood-level SES and fruit consumption in Canada, New Zealand and Scotland, with increased odds of greater fruit intake in higher SES neighbourhoods. In Australia, Canada, New Zealand and Portugal, those residing in higher SES neighbourhoods had increased odds of greater vegetable intake. The other studies showed no evidence of a difference by neighbourhood-level SES.

**Conclusions:**

Acknowledging discrepancies across studies in terms of sampling, measures, and definitions of neighbourhoods, this opportunistic study, which treated data in a consistent manner, suggests that associations between diet and neighbourhood-level socioeconomic status vary across countries. Neighbourhood socioeconomic disadvantage may differentially impact on access to resources in which produce is available in different countries. Neighbourhood environments have the potential to influence behaviour and further research is required to examine the context in which these associations arise.

## Background

Low consumption of fruit and vegetables is a risk factor for poor health [1]. Dietary risk factors account for one tenth of the total global disease burden [2]. Some evidence suggests that fruit and vegetable consumption varies across neighbourhoods, with lower intakes observed amongst more socioeconomically disadvantaged neighbourhoods, even after adjustment for individual-level characteristics of residents [3–5]. This may be due, at least in part, to poorer access in disadvantaged neighbourhoods to stores selling fruits and vegetables [6–9]. However, findings from empirical studies of this issue remain inconsistent [10]. The majority of studies supporting associations between neighbourhood socioeconomic status (SES) and diet have been undertaken in the US [11]. Findings from other countries such as Australia [12, 13], Japan [14], the UK [15], the Netherlands [16, 17] and Portugal [18] are more limited, and existing research has suggested few or inconsistent associations between neighbourhood SES and fruit and vegetable purchasing or consumption in these countries. Such inconsistencies across studies and countries may be attributable to differences in study methodologies, including the measurement of dietary outcomes, or the adjustment for different potential confounding variables.

Alternatively, these discrepant findings may indicate that socio-spatial inequities in diet are context-specific, highlighting a need for international comparisons across contexts. Factors such as the regulation of food marketing, agricultural subsidies, dietary guidelines, levels of poverty, availability of food retailing, social norms, socioeconomic segregation and clustering of food outlets across neighbourhoods vary between nations [10]. However, most studies to date have been conducted within a single city or country, and to our knowledge no such comparison has been undertaken. International comparisons are important in that they help to elucidate the generalizability of findings across nations. There are few examples of international comparative work examining eating behaviours [19–22].

This study aimed to describe variations in fruit and vegetable consumption among adults living in neighbourhoods of varying socioeconomic disadvantage, adjusting for individual socio-demographic variables, across seven countries: Australia, New Zealand, Canada, Netherlands, USA, Scotland, and Portugal. Synthesising data to undertake a single set of analyses enables a closer comparison of heterogeneous datasets than is typically possible when data are analysed and reported in independent studies.

## Methods

This study involved secondary analyses of cross-sectional data from seven existing datasets. Studies included those with study variables assessed in adult (>18 years) samples residing in neighbourhoods which covered different levels of socioeconomic status. Study variables included: an indicator of area/neighbourhood-level SES; individual socio-demographic variables including age, sex and education level; and dietary indicators (fruit and vegetable consumption). The comparative analysis presented in this study was unfunded and the inclusion of data for analysis was pragmatic rather than systematic. Potential studies for inclusion were identified based on literature searches and knowledge and links of the co-authors, and primary authors were approached to gauge interest in participating. Three eligible collaborators approached (from the US, Canada, and Australia) declined to be involved due to lack of capacity, and a fourth (from the US) provided data that could not be included due to the lack of specific questions assessing the dietary outcome variables. Full characteristics of included studies and samples are described in detail elsewhere (see study citations), and summarised in Table 1.

**Table 1:** Descriptions, samples and measures of included studies

Study name, country, year of data collection and citation	Sample n and brief description	Average number of participants per neighbourhood (range)	Neighbourhood definition	Neighbourhood SES measure	Fruit/vegetable consumption measures
SESAW^a^, Australia, 2004 [23]	1555 women aged 18–66 years residing in Melbourne	33 (12–48)	Suburbs (N = 45) within 30 km of the central business district.	Socio Economic Index For Areas (SEIFA).	Number of servings of fruit and vegetables eaten per day.
			The suburbs sampled had an average population size of 11 717 people (range: 2729–45 509) and an average geographic size of 6.34 km^2^ (range: 0.89–30.2)	Suburbs in study area were ranked according to SEIFA score, and 15 suburbs were drawn randomly from each of the lowest, middle and highest SEIFA septiles.	Self-report postal dietary survey.
					Fruit: ‘*How many serves of fruit do you usually eat each day?*’
					Described as one medium piece of two small pieces of fruit, or one cup of diced fruit.
					Vegetables: ‘*How many serves of vegetables do you usually eat each day?*’
					Described as ½ cup of cooked vegetables or 1 cup of salad vegetables. Questions based on those in the Australian National Nutrition Survey.
					Response categories: None; one serve; 2 serves; 3–4 serves; 5 serves or more.
New Zealand Health Survey, 2002/03 [24]	12529 participants aged 15–97 years residing in New Zealand	11 (1–83)	Census meshblocks (N = 1178)	2001 New Zealand Deprivation Index.	Number of servings of fruit and vegetables eaten per day.
	(subsample ≥18 years considered)		Mean population c.100, ranging in size from 0 and 624. Meshblocks ranged in size 1 km^2^-2197 km^2^	All 38,350 meshblocks in NZ were divided into quintiles according the deprivation score.	Self-report nutrition questionnaire as part of the Health Survey.
					Fruit: ‘*On average, how many servings of fruit (fresh, frozen, canned or stewed) do you eat per day?’*
					Excludes fruit juice and dried fruit. A serving = 1 medium piece or 2 small pieces of fruit or ½ cup of stewed fruit. For example, 1 apple and 2 small apricots = 2 servings.
					Vegetables: ‘*On average, how many servings of vegetables (fresh, frozen, or canned) do you eat per day?*’
					Excludes vegetable juice. A serving = 1 medium potato/kumara or ½ cup cooked vegetables or 1 cup of salad vegetables. For example, 2 medium potatoes and ½ cup of peas = 3 servings.
					Response categories:
					I don’t eat vegetables/fruit; less than 1 serving per day; 1 serving per day; 2 servings per day; 3 servings per day; 4 or more servings per day.
PHS Edmonton^b^, Canada, 2001 [25]	4175 participants aged 18–95 years residing in Edmonton	15 (1–95)	Administrative boundaries (N=214)	Neighbourhood SES Index grouped into tertiles which were created across the sample of 4175 participants.	Number of portions of fruit and vegetables eaten per week.
					Telephone administered survey.
			The neighbourhoods sampled had an average population size of 3 042 people (range: 110–15 260, SD= 1 811) and an average geographic size of 1.69 km^2^ (range: 0.21–44.57, SD=4.38)		Fruit: ‘*Not counting juice, how often (number of times per week) do you usually eat fruit?*’
					Vegetables: ‘*How many servings (number of servings per week) of vegetables do you usually eat?*’
					Weekly number of fruit and vegetable portions consumed recorded in separate variables. Average daily amount was calculated from the reported number of each consumed.
GLOBE^c^, Netherlands, 2004 [26]	660 participants aged 25–75 years residing in Eindhoven	47 (16–91)	Administrative boundaries (N=14)	NIVEL deprivation index. Neighbourhoods in the study area were ranked according to the NIVEL score, and fourteen neighbourhoods were drawn: seven among those with the lowest and seven among those with the highest scores.	Amount of fruit and vegetables (in grams) eaten per day.
					Self-report postal survey.
					Fruit:
					1) For several fruit items, participants reported how many times they consumed this item on a weekly/monthly basis, and how many portions they ate on such an occasion. Intakes of each item were calculated by multiplying frequency and portion size. Intakes were summed across the various fruit items to obtain total fruit intake.
					2) Two-item question:
					a) On a day you consume fruit, how many pieces do you eat on average?
					b) On how many days per week do you consume this amount of pieces of fruit?
					Vegetables: Separate report of how many portions hot (cooked/baked) vegetables and cold (salad/lettuce/tomato/cucumber) vegetable intake on a weekly/monthly basis. Intake was calculated by multiplying frequency and portion size and the average vegetable intake per day was calculated.
HEP survey^d^, USA, 2002/03 [27]	919 participants aged 25–96 years residing in three areas of Detroit, Michigan	11 (1–42)	Census block group (N=69)	Median household income from the 2000 Census in tertiles based on the study areas.	Mean number of daily servings of fruit and vegetables.
				Participants were drawn using a stratified sampling design, using six strata defined as follows:	Interviewer-administered, modified Block 98 semi-quantitative Food Frequency Questionnaire (Berkeley Nutrition Services, Berkeley, California)
			The Census block groups from which the sample was drawn had an average population size of 941 people (range: 301–2073) and an average geographic size of 0.15square miles (range: 0.03–0.55).	<20% poverty, <80% African American	Fruit: For 11 items, participants were asked how many times they consumed this item on a weekly/monthly basis and usual portion size.
				<20% poverty, >=80% African American	Vegetables: For 20+ items (including potatoes), participants were asked how many times they consumed the item on a weekly/monthly basis and usual portion size.
				20-<40% poverty, <80% African American	For both fruit and vegetables, intake of each item was calculated by multiplying frequency and portion size. Intakes were summed and average daily intake was calculated.
				20-<40% poverty, >=80% African American	
				>=40% poverty, <80% African American	
				>=40% poverty, >=80% African American	
GGHBHAW^e^, Scotland, 2002 [28]	1802 individuals aged 16–99 years residing in Greater Glasgow	40 (4–108)	Postcode sector (N=121)	Carstairs deprivation score. The HWB sample was stratified proportionately by local authority and deprivation category (DEPCAT), with addresses selected randomly.	Number of portions of fruit or vegetables eaten each day.
	(subsample ≥18 years considered)				Self-report postal survey.
					Fruit:’*On average, how many portions of fruit do you eat EACH DAY? Examples of a portion are one apple, one tomato, 2 tablespoons canned fruit, one small glass fruit juice.*’
					Vegetables: ‘*On average, how many portions of vegetables or salad (not counting potatoes) do you eat EACH DAY? A portion of vegetables is 2 tablespoons.*’
NHS-LMA^f^, Portugal, 1998/99 [18]	7665 individuals aged 18–96 years residing in Lisbon Metropolitan Area	53.6 (19–222)	Administrative boundaries	Composite measure, operationalized following the methodology of Carstairs and Morris (1991). Standardization and sum of three census variables: male unemployment, unskilled worker employment and individuals living in shanty houses. Higher values indicate higher deprivation.	Any fruit or vegetables consumed on the previous day.
			(N=143 parishes) from Lisbon Metropolitan Area. The suburbs sampled had an average population size of 14.825 people (range: 660–61.373) and an average geographic size of 12.45km^2^ (range: 0.05–212.34).		Self-report questionnaire.
					For several food items (soup, fish, meat, potatoes/rice/pasta, vegetables, fruit, bread, and other foods), participants reported if they consumed the item in the day before the survey.
					The questions selected for this study were:
					Fruit: ‘*Did you eat any fruit yesterday?*’
					Vegetables: ‘*Did you eat any vegetables yesterday?*’
					Response categories: Yes, No and I don’t know.
					Potatoes and Soup were not included on the vegetable intake.

^a^SESAW = SocioEconomic Status and Activity in Women study. ^b^PHS Edmonton = Edmonton Population Health Survey. ^c^GLOBE = Health and Living Conditions of the Population of Eindhoven study. ^d^HEP = Healthy Environments Partnership study. ^e^GGHBHAW = Greater Glasgow Health Board Health and Wellbeing survey.^f^NHS-LMA = National Health Survey for the Lisbon Metropolitan Area

Briefly, the studies included were the SocioEconomic Status and Activity in Women (SESAW) study from Australia [23]; the New Zealand Health Survey [24]; the Edmonton Population Health Survey (PHS Edmonton) from Canada [25]; the Health and Living Conditions of the Population of Eindhoven (GLOBE) study from the Netherlands [26]; the Healthy Environments Partnership (HEP) study from the US [27]; the Greater Glasgow Health Board Health and Wellbeing (GGHBHAW) survey from Scotland [28]; and a sub-sample from the National Health Survey for the Lisbon Metropolitan Area, Portugal (NHS-LMA) [18].

### Measures

The measures of neighbourhood SES and fruit and vegetable consumption are summarised in Table 1. All studies included a measure of respondents’ education level, which was used as an indicator of individual socioeconomic position (SEP).

#### Fruit and vegetable consumption

Separate binary indicator variables of fruit and vegetable intake were created for each study. Where possible, for comparability across studies, fruit intake was grouped into categories of <2 or ≥2 serves per day, chosen to correspond to commonly recommended fruit intakes in a number of countries [29–31]. Vegetable intake was considered as <3 or ≥3 serves per day since the samples meeting recommended guidelines (5 serves/day) were too small to allow meaningful comparisons, thus a more liberal criteria was selected. In the NHS-LMA (Portugal), respondents were asked “Did you eat any fruit yesterday?” and “Did you eat any vegetables yesterday?” Therefore, consumption in this study was recorded as ‘yes’ or ‘no’. Whilst this question did not allow categorisation exactly like other countries, it did help to distinguish between those who reported higher fruit and vegetable intake relative to other participants. In GLOBE (Netherlands), consumption was recorded as <250 g or ≥250 g for fruit and <200 g or ≥200 g for vegetables (roughly 3 and 2.5 serves, respectively, based on standard average portion sizes equivalent to 80 g).

#### Neighbourhood-level SES

In six studies, neighbourhood-level SES was grouped into three categories: low, medium and high. In GLOBE, neighbourhood SES was recorded as low or high, since only neighbourhoods from the two extremes were purposively selected to maximise contrast.

The Socio Economic Index for Areas Index of Relative Socio-Economic Disadvantage (SEIFA IRSD) [32] was used as a measure of neighbourhood-level SES in SESAW (Australia). Neighbourhoods (suburbs) were randomly selected from the lowest, middle, and highest septile of SES. In the New Zealand Health Survey, quintiles of the 2001 New Zealand Deprivation Index [33] (ranging from 1 = least deprived to 5 = most deprived) were grouped into three categories of low (quintiles 4 and 5), medium (quintiles 2 and 3) and high (quintile 1) SES. In PHS Edmonton, neighbourhood-level SES was defined as a sum of *z-*scores of net educational level and median income of census families minus the *z* score of the proportion of unemployed [34] (ranging from −7.5 to 9.79) grouped into tertiles of low (≤ − 1.25), medium (> − 1.25 to 0.05) or high (>0.05). Net educational level was obtained by subtracting the proportion of individuals aged ≥15 years with lower education (no diploma, certificate, or degree) from the proportion with higher education (a university diploma, certificate, or degree) in each neighbourhood. In GLOBE, the Netherlands Institute of Research in Healthcare (NIVEL) deprivation index was used to indicate neighbourhood SES (based on the proportion of the population that is economically active, average income, proximity index and proportion of the population who are non-Western foreigners). Fourteen neighbourhoods were selected: seven among the lowest and seven among the highest level of deprivation. In HEP, median household income from the 2000 Census was used to define neighbourhood-level SES. Areas categorized as low, middle and high SES for this sample had median household incomes of ≤ $22,589, >$22,589 to ≤ $27,170, and > $27,107, respectively. The median household income for the US as a whole in 2000 was $42,142 and for the state of Michigan in the same year was $46,181 [35], thus even the high SES areas included in the HEP study were below the median for the US as well as for Michigan. In GGHBHAW, neighbourhood-level SES was represented by the 7-fold Carstairs deprivation categorisation which ranges from 1 = most affluent to 7 = most deprived, regrouped as high (1–2), medium (3–5), or low (6–7). In NHS-LMA, neighbourhood-level deprivation was operationalised following the methodology of Carstairs and Morris [36] (standardization and sum of three census variables: male unemployment, unskilled worker employment and individuals living in shanty houses) split into tertiles of high (<−0.80), medium (−0.76 to <0.47) and low (0.47 to 15.8) SES (higher values indicate higher deprivation).

#### Individual socio-demographic variables

Age (years), gender, height and weight (and calculated body mass index (BMI)) were reported in each study, although by design the SESAW study included only women.

Education level was available for all studies and was grouped into three categories: low, medium and high. The definitions varied slightly by study and were categorised either according to years of schooling or certification obtained. Low education was defined as <12 years of education in both SESAW and HEP, and <11 years in NHS-LMA. For both the New Zealand Health Survey and PHS Edmonton, this category consisted of those with no secondary/high school qualifications. Similarly, those who had completed only primary or lower secondary education in GLOBE and those who had either no education or lower high school qualifications (e.g., Standard Grades or GCSEs) in GGHBHAW were grouped as low education. Medium education across the studies typically indicated those who completed secondary school with a certificate/qualification and/or had a vocational qualification. A high level of education referred to those with a degree or higher degree across all studies, apart from the NHS-LMA study where this category also included those attending university.

### Statistical analysis

With one exception (HEP), statistical analyses for all studies were undertaken by a single statistician (KEL). For the HEP study, protocols prevented data provision and hence analyses were undertaken by a second analyst (GM), in close consultation with KEL. For each study, marginal logistic regression models were fitted to examine the association between neighbourhood-level SES and fruit and vegetable consumption separately, adjusting for the clustering of individuals within neighbourhoods using generalised estimating equations with exchangeable correlation structure and robust standard errors. Unadjusted analysis and analyses adjusted for age, gender, and education level were considered.

## Results

Descriptive characteristics are shown in Table 2. The average age across the seven studies was fairly comparable, at between 42 and 52 years. Between 39 % and 50 % of study participants were male apart from in the SESAW study, designed to only sample women. The US HEP study had the highest average BMI at 30.8 kg/m^2^ and the highest proportion of individuals weighing over 30 kg/m^2^ (48 %). Most other studies had an average BMI of approximately 25 kg/m^2^. SESAW (Australia) had the largest proportion of individuals within the highly educated category (36 %) while the HEP survey had the lowest at only 8 %.

**Table 2:** Descriptive characteristics of the samples by survey

	SESAW^a^	New Zealand Health Survey	PHS Edmonton^b^	GLOBE^c^	HEP^d^	GGHBHAW^e^	NHS-LMA^f^
	(N = 1535)	(N = 12147)	(N = 3189)	(N = 634)	(N = 919)	(N = 1747)	(N = 7665)
	N (%) or Mean (SD) & Range	N (%) or Mean (SD) & Range	N (%) or Mean (SD) & Range	N (%) or Mean (SD) & Range	N (%) or Mean (SD) & Range	N (%) or Mean (SD) & Range	N (%) or Mean (SD) & Range
Response variables							
Fruit intake							
*≥2 serves/day* ^ǂ^ *(all studies except NHS-LMA* ^ǂǂ^ *)*	927 (60.4 %)	6634 (54.6 %)	1318 (41.3 %)	358 (56.5 %)	184 (21.2 %)	931 (53.3 %)	6888 (89.9 %)
Vegetable intake							
*≥3 serves/day* ^†^ *(all studies except NHS-LMA* ^††^ *)*	522 (34.0 %)	7996 (65.8 %)	862 (27.0 %)	138 (21.8 %)	186 (22.4 %)	440 (25.2 %)	6022 (78.6 %)
Key predictor variable							
Neighbourhood SES							
By individual							
*Low*	468 (30.5 %)	7146 (58.8 %)	1093 (34.3 %)	283 (44.6 %)	298 (31.2 %)	871 (49.9 %)	2202 (28.7 %)
*Medium*	572 (37.3 %)	3353 (27.6 %)	1040 (32.6 %)	-	324 (33.8 %)	611 (35.0 %)	2837 (37.0 %)
*High*	495 (32.3 %)	1648 (13.6 %)	1056 (33.1 %)	351 (55.4 %)	297 (34.9 %)	265 (15.2 %)	2626 (34.3 %)
By neighbourhood							
*Low*	15 (33.3 %)	623 (53.0 %)	75 (31.3 %)	7 (50.0 %)	23 (33.3 %)	65 (54.6 %)	47 (32.9 %)
*Medium*	15 (33.3 %)	355 (30.2 %)	65 (27.0 %)	-	19 (27.5 %)	35 (29.4 %)	48 (33.6 %)
*High*	15 (33.3 %)	197 (16.8 %)	101 (41.9 %)	7 (50.0 %)	27 (39.1 %)	19 (16.0 %)	48 (33.6 %)
Socio-demographic variables							
Age (years)	41.7 (12.6)	46.4 (17.4)	43.6 (19.1)	52.2 (13.7)	46.3 (24.3)	52.2 (19.6)	48.5 (18.0)
	18–66	18–97	18–93	25–75	25–96	18–99	18–96
*Missing*	54 (3.5 %)	0 (0.0 %)	28 (0.9 %)	0 (0.0 %)	0 (0.0 %)	18 (1.0 %)	0 (0.0 %)
Gender							
*Male*	-	4704 (38.7 %)	1582 (49.6 %)	287 (45.3 %)	287 (47.7 %)	679 (38.9 %)	3565 (46.5 %)
*Missing*		0 (0.0 %)	0 (0.0 %)	0 (0.0 %)	0 (0.0 %)	2 (0.1 %)	0 (0.0 %)
BMI (kg/m^2^)	24.7 (5.5)	27.9 (6.3)	25.5 (4.7)	25.8 (4.2)	30.8 (6.1)	25.1 (4.4)	25.6 (4.2)
	12.0–58.0	13.5–63.7	14.5–63.2	16.7–56.5	15.8–61.8	13.0–52.5	18.5–58.7
*Missing*	102 (6.6 %)	1187 (9.8 %)	115 (3.6 %)	5 (0.8 %)	0 (0.0 %)	44 (2.5 %)	0 (0.0 %)
BMI category							
*<20 kg/m* ^*2*^	175 (11.4 %)	668 (5.5 %)	261 (8.2 %)	24 (3.8 %)	31 (3.8 %)	144 (8.2 %)	399 (5.2 %)
*20 - <25 kg/m* ^*2*^	680 (44.3 %)	3293 (27.1 %)	1354 (42.5 %)	258 (40.7 %)	163 (17.3 %)	820 (46.9 %)	3392 (44.3 %)
*25 - <30 kg/m* ^*2*^	335 (21.8 %)	3634 (29.9 %)	1023 (32.1 %)	262 (41.3 %)	271 (31.1 %)	537 (30.7 %)	2821 (36.8 %)
*≥30 kg/m* ^*2*^	243 (15.8 %)	3365 (27.7 %)	436 (13.7 %)	85 (13.4 %)	454 (47.9 %)	202 (11.6 %)	1053 (13.7 %)
*Missing*	102 (6.6 %)	1187 (9.8 %)	115 (3.6 %)	5 (0.8 %)	0 (0.0 %)	44 (2.5 %)	0 (0.0 %)
Education level							
*Low*	350 (22.8 %)	3476 (28.6 %)	438 (13.7 %)	300 (47.3 %)	328 (37.0 %)	1078 (61.7 %)	5564 (72.6 %)
*Medium*	602 (39.2 %)	5421 (44.6 %)	1922 (60.3 %)	156 (24.6 %)	512 (54.7 %)	310 (17.7 %)	1034 (13.5 %)
*High*	548 (35.7 %)	3244 (26.7 %)	819 (25.7 %)	178 (28.1 %)	79 (8.3 %)	340 (19.5 %)	1062 (13.9 %)
*Missing*	35 (2.3 %)	6 (0.1 %)	10 (0.3 %)	0 (0.0 %)	0 (0.0 %)	19 (1.1 %)	5 (0.1 %)

*Sample sizes omitting those with missing fruit and vegetable consumption and neighbourhood SES

ǂGLOBE: ≥250 g/day; ǂǂ Portugal: any fruit yesterday- yes. † GLOBE: ≥200 g/day; †† Portugal: any vegetables yesterday- yes

^a^SESAW = SocioEconomic Status and Activity in Women study. ^b^PHS Edmonton = Edmonton Population Health Survey. ^c^GLOBE = Health and Living Conditions of the Population of Eindhoven study. ^d^HEP = Healthy Environments Partnership study. ^e^GGHBHAW = Greater Glasgow Health Board Health and Wellbeing survey.^f^NHS-LMA = National Health Survey for the Lisbon Metropolitan Area

Fruit intake was lowest in the HEP survey (US); only 21 % of participants consumed at least 2 serves per day. The highest consumption of fruit was in the NHS-LMA study (Portugal) (90 %), although this study only contained information about any consumption the day previously. Similarly, the NHS-LMA study had the highest level of vegetable consumption (79 %), as this question did not ask about the quantity of vegetables consumed. We observed the lowest prevalence of eating adequate amounts of vegetables in the Dutch and US study; only 22 % within each sample consumed at least 3 serves (although in GLOBE the question was phrased in terms of grams consumed, i.e., ≥200 g/day or around 2.5 serves).

### Fruit consumption

In unadjusted analyses, there was evidence of an association between neighbourhood-level SES and fruit consumption for four studies: SESAW (Australia), the New Zealand Health Survey, PHS Edmonton (Canada), and GGHBHAW (Scotland) (Table 3). In each of these studies the odds of eating ≥2 serves of fruit daily increased with increasing neighbourhood-level SES. These results held after adjustment for education and other socio-demographic variables for all studies apart from SESAW. There was no evidence of an association between neighbourhood-level SES and fruit intake for GLOBE, HEP or NHS-LMA.

**Table 3:** Unadjusted and adjusted marginal logistic regression models of the odds of consuming at least 2 serves of fruit or 3 serves of vegetables per day by neighbourhood SES*

	SESAW^a^	New Zealand Health Survey	PHS Edmonton^b^	GLOBE^c^	HEP^d^	GGHBHAW^e^	NHS-LMA^f^
	(N = 1458)	(N = 12141)	(N = 3153)	(N = 634)	(N = 919)	(N = 1711)	(N = 7660)
Fruit intake	OR (95 % CI)	OR (95 % CI)	OR (95 % CI)	OR (95 % CI)	OR (95 % CI)	OR (95 % CI)	OR (95 % CI)
*Unadjusted*							
Neighbourhood SES							
*Medium*	**1.35 (1.00, 1.82)**	**1.41 (1.28, 1.56)**	1.10 (0.91, 1.34)	-	1.10 (0.77, 1.58)	**1.79 (1.29, 2.48)**	1.03 (0.74, 1.41)
*High*	**1.48 (1.11, 1.98)**	**1.61 (1.41, 1.83)**	**1.31 (1.11, 1.53)**	1.10 (0.81, 1.48)	1.17 (0.81, 1.67)	**2.71 (1.85, 3.96)**	0.96 (0.71, 1.30)
*Adjusted*							
Neighbourhood SES							
*Medium*	1.18 (0.87, 1.60)	**1.29 (1.16, 1.43)**	1.05 (0.86, 1.28)	-	1.09 (0.76, 1.56)	**1.61 (1.17, 2.23)**	0.98 (0.69, 1.38)
*High*	1.10 (0.80, 1.50)	**1.42 (1.25, 1.62)**	**1.21 (1.03, 1.42)**	1.05 (0.74, 1.47)	1.17 (0.81, 1.68)	**2.07 (1.39, 3.09)**	0.93 (0.68, 1.27)
Vegetable intake	**OR (95 % CI)**	**OR (95 % CI)**	**OR (95 % CI)**	**OR (95 % CI)**	**OR (95 % CI)**	**OR (95 % CI)**	**OR (95 % CI)**
*Unadjusted*							
Neighbourhood SES							
*Medium*	**1.94 (1.36, 2.75)**	**1.53 (1.35, 1.73)**	**1.32 (1.07, 1.63)**	-	1.43 (0.99, 2.04)	0.93 (0.59, 1.46)	**1.62 (1.21, 2.17)**
*High*	**2.17 (1.56, 3.01)**	**1.57 (1.33, 1.85)**	**1.51 (1.24, 1.83)**	1.31 (0.83, 2.08)	1.19 (0.81, 1.74)	1.00 (0.54, 1.87)	**1.75 (1.31, 2.33)**
*Adjusted*							
Neighbourhood SES							
*Medium*	**1.79 (1.26, 2.53)**	**1.41 (1.25, 1.60)**	1.19 (0.96, 1.48)	-	1.41 (0.98, 2.02)	0.88 (0.56, 1.39)	**1.56 (1.14, 2.13)**
*High*	**1.74 (1.22, 2.48)**	**1.44 (1.22, 1.70)**	**1.29 (1.06, 1.57)**	1.21 (0.71, 2.06)	1.18 (0.80, 1.73)	0.97 (0.52, 1.79)	**1.68 (1.25, 2.62)**

Results in bold indicate p < 0.05

*Sample sizes omit those with missing data across all variables

^a^SESAW = SocioEconomic Status and Activity in Women study. ^b^PHS Edmonton = Edmonton Population Health Survey. ^c^GLOBE = Health and Living Conditions of the Population of Eindhoven study. ^d^HEP = Healthy Environments Partnership study. ^e^GGHBHAW = Greater Glasgow Health Board Health and Wellbeing survey.^f^NHS-LMA = National Health Survey for the Lisbon Metropolitan Area

#### Vegetable consumption

The odds of eating ≥3 portions of vegetables increased with increasing neighbourhood-level SES in SESAW (Australia), the New Zealand Health Survey, and PHS Edmonton (Canada) (Table 3). These results held after adjustment for other socio-demographic variables. In addition, there was evidence that the odds of eating any vegetables on the previous day increased with increasing SES in NHS-LMA (Portugal). For HEP (US), the odds of consuming ≥3 portions of vegetables were somewhat higher among residents of medium SES neighbourhoods compared to residents in low SES neighbourhoods in unadjusted and adjusted analyses (p < 0.10). There was no evidence of an association between SES and vegetable intake for GLOBE (Netherlands) or GGHBHAW (Scotland).

## Conclusions

To the authors’ knowledge, this paper represents the first attempt, using secondary data analysis, to undertake an examination of variations in neighbourhood disadvantage and fruit and vegetable consumption in multiple countries. The study was unfunded and opportunistic, and inevitably there were discrepancies across the studies in terms of sampling, measures, and definitions of neighbourhoods. Consequently it was not possible to pool data for a meta-analytic or pooled analytical approach. However the comparison was intentionally limited to studies that were as comparable as possible across countries, and where similar variables were available. It was not possible to draw the same conclusions from previously published data alone, since existing published papers from the studies included did not treat variables in a consistent manner, nor control for the same confounders. Therefore, acknowledging its limitations and particularly the inability to draw strong conclusions as to the comparative magnitude of effects across studies, the paper represents a novel contribution to the existing literature on neighbourhood deprivation and fruit and vegetable consumption across developed countries.

The present preliminary findings showed that, after considering key confounding variables, of the seven studies included, neighbourhood-level SES was positively associated with residents’ fruit consumption in three studies (New Zealand; Edmonton, Canada; and Glasgow, Scotland), and vegetable consumption in four studies (New Zealand; Edmonton, Canada; Melbourne, Australia; and Lisbon, Portugal), with weak evidence of an association in a fifth (US HEP survey). The remaining studies showed no associations between area SES with fruit or vegetable intake in this exploratory analysis. These discrepant findings across studies, despite attempts in analyses to match the outcome and confounding variables as closely as possible, suggest that there may exist true differences in the associations of neighbourhood disadvantage with fruit and vegetable consumption across localities. While we cannot rule out that the differences are attributable to other methodological inconsistencies across datasets (for example, in the ways in which ‘neighbourhoods’ or area socioeconomic status were defined, or the ways in which sampling was undertaken), the results nonetheless highlight the importance of considering context in investigations of area-level deprivation and dietary outcomes. Generalizing findings from one study or context to another may be inappropriate and may lead to erroneous conclusions about the potential role of neighbourhoods in diet.

The findings of null associations of area SES with fruit and/or vegetable intake in adjusted analyses in the GLOBE and NHS-LMA studies (and borderline association in the HEP Study) could be attributable to several factors. Firstly, the GLOBE and the HEP studies had the smallest sample sizes, and it is possible that these were not sufficient to detect associations of small magnitudes. However, the large sample size of the NHS-LMA study suggests this was not likely to be the case here. Secondly, the NHS-LMA study used a relatively crude indicator of fruit and vegetable intake, with the majority of participants (90 % and 79 %, respectively) scoring above the cut-points, which may have contributed to reduced sensitivity to detect associations. Thirdly, in the HEP survey, all of the study neighbourhoods fell below the national mean in terms of neighbourhood SES. Differences in dietary intakes across these neighbourhoods may hence have been less pronounced than they might have been in a sample with a broader range of neighbourhood SES.

Alternatively, however, it may be that the inconsistency of associations observed across studies reflects cross-country differences in the social, built, economic, or regulatory environmental factors that influence fruit and vegetable provision, purchase, and consumption [10]. Socioeconomic residential segregation may be more pronounced, and food planning regulations less focused on compensating for such segregation, in countries like the US and Canada than in the UK, Europe, or Australia [10]. For example, the Netherlands is a densely populated country, with a high density of fresh food outlets (e.g., the average distance to a supermarket in 2012 was 900 m [37]), and the accessibility of fruit and vegetables is hence relatively good for residents of both low and high SES neighbourhoods [38]. In Glasgow, supermarkets have been found to be more prevalent in poorer areas, possibly due to regulatory controls and lower land prices [39]. Supermarket restrictive covenants (private legal agreements imposed on former supermarket sites) currently limit the use of 18 sites – particularly in socioeconomically disadvantaged neighbourhoods – for food sales in Edmonton, Canada [40], effectively reducing access to healthy foods for local residents and possibly exacerbating neighbourhood inequities in healthy food consumption. Such contextual differences are consistent with the findings of null associations of neighbourhood deprivation with fruit and/or vegetable intakes in studies such as the GLOBE (Netherlands), NHS-LMA (Portugal) and GGHBHAW (Scotland) compared with positive associations in others including PHS (Edmonton).

Our results showed, however, that in studies where evidence of an association was observed, these associations were in the expected direction – that is, residents of more advantaged neighbourhoods had greater odds of consuming fruits and vegetables. These findings are generally consistent with those of a systematic review [41] that assessed links between SES and fruit and vegetable consumption in adults, finding substantial evidence of positive associations. However, that review was limited to European studies, and reported on associations from published studies which did not attempt to undertake comparable analyses. In addition, the indicators of SES in that study were primarily those at the individual level; while ‘other’ indicators were described, these included a combination of less typical individual-level characteristics (e.g., car ownership) as well as area-based measures, making conclusions regarding area deprivation and diet alone difficult. Results from studies outside of Europe (not represented in that review) have also shown increased fruit and vegetable consumption with increasing neighbourhood-level socioeconomic position [3, 42].

A number of limitations of this study should be acknowledged. In addition to the issues of varying comparability described above, not all samples were nationally representative. Furthermore, included studies were not selected using a systematic approach so the findings from these studies may not be representative. The timing of the surveys also varied, although not vastly (1998–2004), and there is no strong reason to expect major changes in general direction of associations to have occurred during that time. The studies used differing methods to assess fruit and vegetable consumption, with some requiring respondents to recall intake per day, another on the previous day, and another per week, for example. In addition, the studies used different spatial scales to assess ‘neighbourhood’, and neighbourhoods incorporated varying numbers of participants, as noted in Table 1. The range of socioeconomic status encompassed in the samples also varied, with for example, the HEP study conducted in predominantly low to moderate income communities, while others reflected a broader range of socioeconomic characteristics. Education was used to represent individual socio-economic position in the adjusted models for each study due to the fact that each study collected data on this variable, and it was possible to treat the measures of education used across studies in a consistent manner. It is not known whether results would have differed if other measures of individual socioeconomic position, such as occupation or income, had been available and able to be adjusted for. The cross-sectional design precludes conclusions regarding causality.

Despite these limitations, the opportunity to integrate and treat existing data in a consistent manner to investigate associations using a single analytical approach across datasets represents an advance over current literature, providing greater comparability across datasets and localities than is currently possible drawing only on separate published studies. Future purpose-designed studies including nationally representative samples, where possible, from a wide range of sources involving a-priori matching on study design, sampling and operationalization and measurement of indicators of SES and diet will provide even more certainty regarding the context-specific nature of the associations of neighbourhood disadvantage and diet. Should future findings confirm associations of neighbourhood socioeconomic conditions with fruit and vegetable intake in particular contexts, further investigation of the drivers of such associations within each context would be important for identifying potential environmental and policy actions to help redress socioeconomic inequalities in these important dietary components.

## Abbreviations

SES: Socioeconomic status
SEP: Socioeconomic position
